# Solvent Selective Effect Occurs in Iodinated Adamantanone Ferroelectrics

**DOI:** 10.1002/advs.202201702

**Published:** 2022-04-25

**Authors:** Lei Xu, Yao Zhang, Huan‐Huan Jiang, Nan Zhang, Ren‐Gen Xiong, Han‐Yue Zhang

**Affiliations:** ^1^ Jiangsu Key Laboratory for Science and Applications of Molecular Ferroelectrics Southeast University Nanjing 211189 P. R. China; ^2^ State Key Laboratory of Bioelectronics School of Biological Science and Medical Engineering Southeast University Nanjing 210096 P. R. China

**Keywords:** ferroelectrics, halogen substitution, physical performance optimization, polymorphs, solvent selective effect

## Abstract

Organic ferroelectrics, as a type of crystalline compound, are generally solution processing. However, for most crystalline compounds, the changing of solvent would not influence the crystalline phase, let alone their physical performance. Here, the solvent selective effect occurs in the iodinated adamantanone ferroelectrics. By changing the solvent with different polarities, the ferroelectric crystals can be induced in two different phases, which is unprecedented to the knowledge. More strikingly, this solvent‐induced transformation could realize the physical performance optimization in the orthorhombic phase (*orth*‐I‐OA, obtained from ethanol) with a stronger second harmonic generation (SHG) response, greater piezoelectric coefficient *d*_33_ of 5 pC N^−1^, and larger spontaneous polarization (*P*_s_) of 3.43 µC cm^−2^ than those of monoclinic one (*mono*‐I‐OA, obtained from ethyl acetate). Such an intriguing phenomenon might be closely related to solvent polarity. Based on the quantitative and qualitative analyses, the similar interaction energies of these two phases suggest that their transformation could be easily realized via changing the solvent. This work provides new insights into the chemical design and performance optimization of organic ferroelectrics.

## Graphical abstract

By introducing halogen atoms to the spherical 2‐adamantanone (OA) molecule, one systematically synthesizes a group of halogen substituted compounds and obtains an organic ferroelectric 5‐iodo‐2‐adamantanone (I‐OA), and their physical properties such as second harmonic generation, piezoelectricity, and ferroelectricity could be rationally optimized through the solvent selective effect.

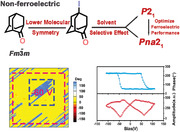

## 1 Introduction

Ferroelectrics are a type of special dielectrics, which have spontaneous polarization (*P*_s_) and their *P*_s_ can be reversed under the external electric field.^[^ ^1^, ^2^, ^3^ ^]^ Combined with their excellent pyroelectric, piezoelectric, and other physical properties, ferroelectrics represented by the inorganic ceramics such as barium titanate (BTO) and lead zirconate titanate (PZT) have been widely used for electronic applications, such as transducers, memories, actuators, and sensors.^[^ ^4^, ^5^, ^6^, ^7^ ^]^ Facing the challenges of the environment, energy, and cost, the focus of materials science has gradually but firmly shifted from inorganic and organic–inorganic systems to organic ones.^[^ ^8^ ^]^ Recently, interest in organic ferroelectrics is increasing because of their superiority of lightweight, good degradability, easy solution processing, low acoustic impedance (matches with human bodies), satisfying biocompatibility, and environmental friendliness.^[^ ^9^, ^10^ ^]^ Thus, they are becoming promising candidates in microrobotics, flexible/wearable electronic devices, implanted biomedical devices.^[^ ^11^, ^12^, ^13^ ^]^ Ferroelectrics are generally crystalline compounds, which should crystalize in one of 10 polar point groups, namely, 1 (*C*_1_), 2 (*C*_2_), *m* (*C_s_*), *mm*2 (*C*_2v_), 3 (*C*_3_), 3*m* (*C*_3v_), 4 (*C*_4_), 4*mm* (*C*_4v_), 6 (*C*_6_), 6*mm* (*C*_6v_).^[^ ^14^, ^15^ ^]^ From the perspective of crystallography, crystal symmetry is of great importance to inducing ferroelectricity, which is mainly determined by the molecular packing in the crystal lattice.^[^ ^16^ ^]^ Notably, crystal packing is sensitive to some external stimuli such as crystallizing solvents, pressure, and thermal, which may result in the formation of polymorphs.^[^ ^17^, ^18^, ^19^ ^]^ Polymorphism, the property of solid materials to exist in two or more crystalline forms with different conformations or orientations of the components in the crystal lattice, is a common phenomenon in organic crystals.^[^ ^20^ ^]^ This phenomenon has also been widely utilized in the industries of chemical engineering and pharmaceutical development.^[^ ^21^ ^]^ Generally, polymorphs show markedly different physical properties, which provides a rational method for performance optimization. Within this context, this raises the question of whether we could use some external stimuli to achieve the physical performance optimization in organic ferroelectrics by modulating their crystal phases?

Organic ferroelectrics are generally solution processing which is easy and environment‐friendly in comparison with inorganic ceramics. As a dissolving medium for solutes, the selected solvent plays a crucial role in crystallization, which may render the difference in crystal structures and phases.^[^ ^22^ ^]^ It is known that the auxiliaries such as the solvent and additive would influence the nucleation and growth processes of organic crystals, and their functions could be divided into two categories, that is, inhibitors and promoters. Among them, the auxiliaries usually act as the inhibitors under the circumstances of morphological engineering and etching, crystal symmetry reducing, the absolute structure of chiral molecules and polar crystals assigning, the effect of solvent on crystal growth elucidating, and the desired polymorph crystallizing. The possible mechanism of the selected solvent that would contribute to the formation of a specific crystal phase has been fully explained by Berkovitch‐Yellin et al.^[^ ^23^, ^24^ ^]^ Due to the preferential absorption of different solvent molecules, it would attach to specific crystal faces, and thereby form a solvation layer to prevent the deposition of the solute molecules. In this way, the crystal phases and the corresponding physical properties of organic crystals could also be influenced by changing the solvent. This makes possible the precise design and optimization of organic ferroelectrics through solvent selective effect, which is unprecedented in inorganic ceramics. For example, Fu et al. have reported the ferroic properties in anilinium bromide where the ferroelectricity and ferroelasticity could be transformed through solvent selective effect.^[^ ^25^ ^]^ Although great efforts have been made, the discovered ferroelectric compounds which are solvent‐sensitive still remain very sparse.

**Scheme 1:**
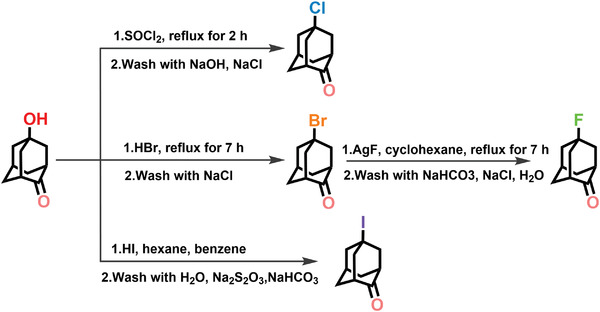
The synthesis processes of F‐OA, Cl‐OA, Br‐OA and I‐OA.

**Scheme 2:**
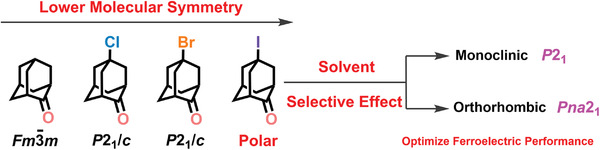
The chemical design of the organic ferroelectric I‐OA through lowering molecular symmetry and its performance optimization by solvent selective effect.

Inspired by the lowering molecular symmetry, we select the spherical molecule 2‐adamantanone (OA) as the building block which possesses a cubic centrosymmetric plastic phase at room temperature and undergoes a structural phase transition at low‐temperature region.^[^ ^26^, ^27^ ^]^ To lower its molecular symmetry, the introducing of a halogen atom should be taken into consideration toward the OA molecule at its 5‐site, going from F to I, because this would also induce a molecular dipole moment. Thus, we obtained the resultant halogen‐substituted compounds through the systematically organic synthesis from the 5‐hydroxy‐2‐adamantanone (**Scheme** **1**). Accompanied by the introducing of halogen atoms on the molecule from F to I, their crystal symmetry is lowered from a centrosymmetric cubic phase to a noncentrosymmetric cubic phase to a centrosymmetric monoclinic phase and finally to a polar monoclinic phase (**Scheme** **2**). It should be highlighted that the iodinated organic ferroelectric 5‐iodo‐2‐adamantanone (*mono‐*I‐OA) is obtained from some solvents with low polarity represented by the ethyl acetate (EA), and it crystallizes in the monoclinic polar space group *P*2_1_ at room temperature. The *mono‐*I‐OA shows a relatively weak ferroelectric performance, such as the second harmonic generation (SHG) intensity of 0.33, piezoelectric coefficient *d*_33_ of 2.1 pC N^−1^, and spontaneous polarization (*P*_s_) of 0.04 µC cm^−2^. Surprisingly, we found another kind of crystal *orth*‐I‐OA which can be obtained in other solvents with high polarity represented by the ethanol. The *orth*‐I‐OA crystal crystallizes in the orthorhombic system with a polar space group of *Pna*2_1_ (point group *mm*2) and possesses optimized physical properties, namely, the accessible SHG response (0.63), the enhanced *d*_33_ (5 pC N^−1^), and the satisfying *P*_s_ (3.43 µC cm^−2^). We speculate that the crystal phase of I‐OA might be dependent on the polarity of the solvent (which could be quantified through their dielectric constant), and a kinetic mechanism instead of a thermodynamic effect might be proposed, indicating that solvent with different polarity shows selective affinity to certain faces and further determine the formation of a particular crystalline phase. Besides, the interaction energy calculation also proved that not the thermodynamic effect but a kinetic mechanism may be responsible for the selective behavior of a solvent. We believe that this work would shed light on the chemical design of organic ferroelectrics as well as their performance optimization.

## 2 Results and Discussion

As a spherical molecule that is similar to [Me_4_N]^+^ and 1,4‐diazabicyclo [2.2.2]octonium (dabco),^[^ ^6^, ^28^, ^29^ ^]^ OA has large conformational freedom which is easier to undergo dynamic rotation and orientational disorder. OA belongs to a highly symmetrical cubic plastic crystal phase at room temperature, and thus we failed to obtain its single crystal structure due to its poor diffraction. In order to reduce the molecular symmetry of the OA molecule, the halogen atoms are added at the 5‐site to induce the molecular dipole moment and asymmetry.

Specifically, the addition of an F atom could smoothly lower down the crystal symmetry into a non‐centrosymmetric space group *F* $\bar{4}$3*m* at 293 K; however, because the F atom has the close van der Waals radius and the similar steric parameters upon the H atom, the crystal symmetry would not largely change and thus lead to the maintaining of the cubic phase. As expected, in comparison with the high‐symmetric cubic plastic phase in OA, the synthesized Cl‐OA and Br‐OA both crystalize in the monoclinic centrosymmetric space group *P*2_1_/*c* at 298 K. Overall, all of them forbid piezoelectricity let alone ferroelectricity. Fortunately, by introducing an iodine atom to the spherical OA molecule, the obtained *mono*‐I‐OA (crystallized from ethyl acetate, petroleum ether, cyclohexane, toluene, ether, dichloromethane, acetone, and acetonitrile) adopts a low‐symmetric polar monoclinic space group *P*2_1_, which belongs to one of the 10 polar point groups *C*_2_ and is allowable for ferroelectricity (Table S1, Supporting Information). Thus, the introducing of a halogen atom on the OA molecule not only lowers the molecular symmetry but also successfully induces a low‐symmetric ferroelectric phase. Besides, by changing the solvent, we obtained another crystal form of I‐OA (*orth*‐I‐OA) by slow evaporation from various solvents such as ethanol, methanol, DMF, and DMSO. The *orth*‐I‐OA crystal crystallizes in the orthorhombic polar *Pna*2_1_ space group (Table S2, Supporting Information).

**Figure 1:**
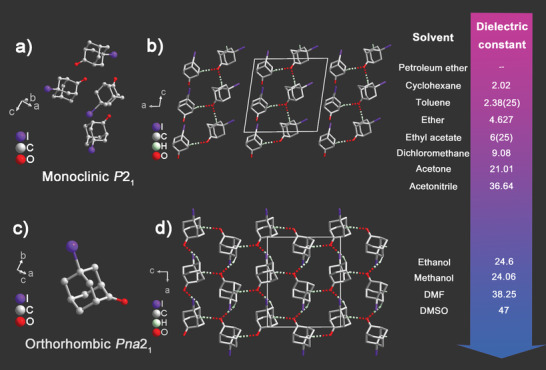
Crystal structures of *mono*‐ and *orth*‐I‐OA crystals obtained from 12 solvents. a,c) The basic unit of *mono*‐ and *orth*‐I‐OA. b,d) The packing view of *mono*‐ and *orth*‐I‐OA along the *b*‐axis at 298 K. The two‐colored dashed lines refer to the weak C—H···O—C molecular interactions of compound *mono*‐ and *orth*‐I‐OA. The dielectric constants are provided for each solvent.^[^ ^33^ ^]^ H atoms were omitted for clarity.

Then, we determined the crystal structures of F‐OA, Cl‐OA, Br‐OA, and I‐OA through the single‐crystal X‐ray diffraction at 293 and 298 K to understand this chemical design strategy of lowering molecular symmetry via the introducing of halogen atoms. The highly disordered state of F‐OA at room temperature makes it behave like a spherical molecule, and the packing view of F‐OA can be seen in Figure S1a,b, Supporting Information. The asymmetric units of Cl‐OA, Br‐OA, and *orth*‐I‐OA are consisting of one respective molecule; while for the *mono*‐I‐OA, its asymmetric unit contains four molecules (**Figure** **1**a,c and Figure S1c,e, Supporting Information). As shown in Figure 1 and Figure S1 (Supporting Information), the corresponding molecules in Cl‐OA, Br‐OA, *mono*‐I‐OA, and *orth*‐I‐OA are ordered and their bond lengths and angles are in the normal range. Structurally, Cl‐OA and Br‐OA are isostructural. Each Cl‐OA and Br‐OA molecule links with the neighbor one through the weak C—H···O—C molecular interactions forming a chain structure along the *c*‐axis, respectively (Figure S1d,f, Supporting Information). The weak C—H···O—C molecular interactions of Cl‐OA and Br‐OA range from 2.594 to 2.687 Å and 2.606 to 2.711 Å shown with the two‐colored dashed lines, respectively. More interestingly, two neighbor parallel chains in the Br‐OA compound are linked by additional halogen···halogen interaction (3.611 Å, marked with yellow dashed lines) between the Br atoms, resulting in a net structure (Figure S1f, Supporting Information). Resembling the Cl‐OA crystal, *mono*‐I‐OA possesses an infinite chain structure along the *c*‐axis through the weak C—H···O—C molecular interactions, while *orth*‐I‐OA forms a net structure (Figure 1b,d). With regard to the *mono*‐ and *orth*‐I‐OA, the weak C—H···O—C interactions are in the range of 2.531 to 2.709 Å and 2.473 to 2.691 Å, respectively. In comparison with the Cl and Br atoms, the I atom is sufficiently large and heavy to freeze the rotational and tumbling motion of this modified molecule which may influence the crystal packing and thus induce the *mono*‐I‐OA to crystallize in a low‐symmetric polar structure. It is noteworthy that I‐OA can attain two polymorphic forms by crystallizing from different solvents. The crystallization process generally consists of two major parts: nucleation and crystal growth. By exploring 12 different solvents ranging in polarity which could be quantified by the dielectric constant from petroleum ether to DMSO, the crystallization processes especially the nucleation and crystal growth processes were controlled, and this may contribute to the turning of the crystal phase of I‐OA changed from *mono*‐I‐OA to *orth*‐I‐OA (except for the acetonitrile). It is difficult to experimentally prove this, while we rationally speculate that solvent molecules would selectively adsorb to particular faces of the growing crystal and retard the growth of the affected faces whilst the crystal continues to grow in the other directions.

Generally, the intermolecular interactions play a critical role in determining the crystal symmetry and inducing inspiring physical properties. Therefore, the Hirshfeld *d*_norm_ surfaces and 2D fingerprint plots are key tools for elucidating the density of intermolecular contacts in one crystal.^[^ ^30^, ^31^ ^]^ As shown in Figure S2 (Supporting Information), both Hirshfeld *d*_norm_ surfaces and 2D fingerprint plots of Cl‐OA, Br‐OA, *mono*‐I‐OA, and *orth*‐I‐OA were calculated and analyzed to clarify the effect of intermolecular interactions. We decomposed the fingerprint plots of the crystal structure of Cl‐OA, Br‐OA, *mono*‐I‐OA, and *orth*‐I‐OA to highlight the particular close contacts for analyses (Figures S3–S6, Supporting Information). As shown in Figure S2a,c (Supporting Information), the interactions between the Cl‐OA and Br‐OA molecules can be seen in the Hirshfeld *d*_norm_ surfaces as the red spots, revealing the C—H_inside_···O and O_inside_···C—H weak interactions between respective molecules. Meanwhile, the red spots are shown in Figure S2c (Supporting Information), indicating the weak halogen···halogen interaction in Br‐OA between the Br···Br atoms. Similar kinds of weak interactions can be found in I‐OA. For *mono*‐I‐OA, each molecule inside the Hirshfeld *d*_norm_ surfaces interacts with one molecule outside the Hirshfeld *d*_norm_ surfaces, forming a chain structure; while for *orth*‐I‐OA, each molecule inside interacts with two molecules outside, supporting the formation of a net structure (Figure S2e,g, Supporting Information). Such a slight distinction in intermolecular interactions may cause a total great difference in their packing structures. The figures of different interactions in the molecules of Cl‐OA, Br‐OA, *mono*‐I‐OA, and *orth*‐I‐OA show comparable differences, suggesting that the contacts around the H atom are the primary ones than the intermolecular interactions which originate from the other atoms as shown in Figures S3–S6, Supporting Information.^[^ ^32^ ^]^

**Figure 2:**
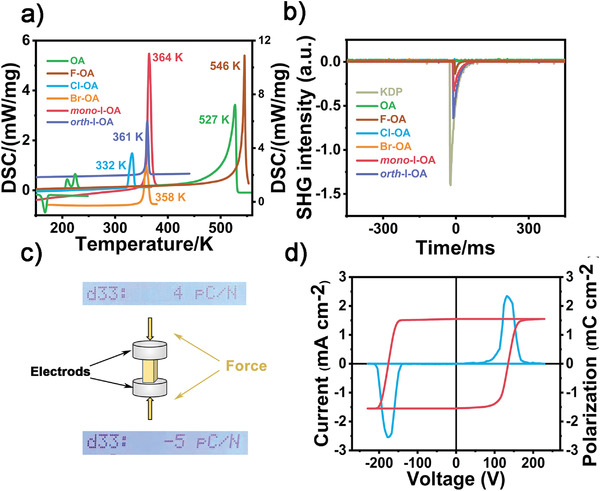
a) DSC curves of OA, F‐OA, Cl‐OA, Br‐OA, *mono*‐I‐OA, and *orth*‐I‐OA. b) SHG intensity of KDP, OA, F‐OA, Cl‐OA, Br‐OA, *mono*‐I‐OA, and *orth*‐I‐OA at *λ* = 1064 nm at room temperature. c) The diagrams of the measured piezoelectric coefficients through the quasi‐static method of crystal sample of *orth*‐I‐OA. d) *P*–*E* hysteresis loop of *orth*‐I‐OA measured at 298 K by using the double‐wave method.

The phase purities of OA, F‐OA, Cl‐OA, Br‐OA, and I‐OA were confirmed by the powder X‐ray diffraction (PXRD) measurements (Figures S7 and S8, Supporting Information) and the ^1^H nuclear magnetic resonance spectroscopy (the details are described in the section of the Experimental section). Combined with the PXRD patterns, the infrared spectra of *mono*‐ and *orth*‐I‐OA (Figure S9, Supporting Information) verify the polymorphism found in the *mono*‐ and *orth*‐I‐OA crystals, which indicates that they are two different types of crystal structures for one I‐OA compound. The thermal stabilities were detected by thermal gravimetric analysis (TGA) measurements, which reveals that OA, F‐OA, Cl‐OA, Br‐OA, *mono*‐I‐OA, and *orth*‐I‐OA are thermally stable until 380, 405, 379, 397, 404, and 400 K, respectively. (Figures S10 and S11, Supporting Information). Differential scanning calorimetry (DSC) measurements were performed to investigate the thermodynamic structural phase transition for OA, F‐OA, Cl‐OA, Br‐OA, *mono*‐I‐OA and *orth*‐I‐OA, as shown in **Figure** **2**a. During the heating process of the DSC measurement, the OA shows two continuous phase transitions at the temperature of 209 and 224 K, and there is only one peak at 166 K in the cooling process. The sum of the entropy changes (Δ*H*) of these two split thermal anomalies in the heating process is almost equal to that of in the cooling process. Negrier et al. have reported the coexistence of both *P*2_1_/*c* and *Cmc*2_1_ phases in the temperature range of 178 to 221 K.^[^ ^27^ ^]^ The variable‐temperature PXRD measurement also confirms this phase transition properties of OA (Figure S12, Supporting Information). Obviously, F‐OA, Cl‐OA, Br‐OA, *mono*‐I‐OA, and *orth*‐I‐OA show no thermodynamic structural phase transitions before their melting point of 546, 332, 358, 364, and 361 K, respectively. The temperature‐dependent real part (*ε′*) of complex dielectric permittivity was also measured to investigate their phase transition behaviors. The temperature‐dependent dielectric constant of OA was measured from 140 to 300 K at a frequency of 1 MHz (Figure S13, Supporting Information). It shows an obvious step‐like dielectric anomaly at 207/171 K, indicating the reversible phase transition of OA. For the other three compounds, no obvious dielectric anomalies can be found, which indicates the absence of structural phase transition before their melting points, in good accordance with their DSC results. However, based on the phase transition properties of OA, we could speculate that their phase transitions might occur after the melting process, in other words, their phase transition temperatures are above their melting points. It is worth noting that the ferroelectricity of *mono*‐ and *orth*‐I‐OA can persist up to 364 and 361 K, and such ferroelectric temperature windows are promising for great application prospects.

The OA, Cl‐OA, and Br‐OA belong to the centrosymmetric space groups. Differently, F‐OA, *mono*‐ and *orth*‐I‐OA crystalize in noncentrosymmetric space groups *F* $\bar{4}$3*m*, *P*2_1,_ and *Pna*2_1_, respectively. Generally, SHG measurement is a powerful tool for detecting noncentrosymmetric compounds because it is sensitive to the absence of inversion center symmetry.^[^ ^34^ ^]^ As shown in Figure 2b, OA, Cl‐OA, and Br‐OA are SHG‐inactive at room temperature. The SHG intensity of F‐OA is about 0.14. *Mono*‐I‐OA is also SHG‐active with the SHG intensity of 0.33 (the SHG intensity is about one‐fourth of that of KH_2_PO_4_ (KDP)). It should be noted that after changing solvent, the *orth*‐I‐OA crystal was obtained, whose SHG intensity nearly doubled to 0.63 in comparison with the *mono*‐phase. The non‐zero SHG responses of *mono*‐ and *orth*‐I‐OA confirm their noncentrosymmetric crystal structures.

Piezoelectric materials generate electrical signals when exposed to mechanical strain in response to an external electric field. Because of the lacking of an inversion center, the noncentrosymmetric compounds are naturally endowed with piezoelectricity. In order to evaluate the piezoelectric coefficient *d*_33_ of *mono*‐ and *orth*‐I‐OA, we used the quasi‐static method and the piezoresponse force microscope (PFM) technique to measure their piezoelectric performance, respectively (Figure 2c and Figure S14d, Supporting Information).^[^ ^35^ ^]^ Considering the point group 2 of *mono*‐I‐OA in its room‐temperature phase, the piezoelectric constant matrix [*d*] can be written as:^[^ ^36^ ^]^

(1) $$\left[\begin{array}{cccccc} 0 & 0 & 0 & d_{14} & 0 & d_{16}\\ d_{21} & d_{22} & d_{23} & 0 & d_{25} & 0\\ 0 & 0 & 0 & d_{34} & 0 & d_{36} \end{array}\right]$$

Here, the piezoelectric performance of *mono*‐I‐OA was determined by the PFM measurement on its single crystal along the *b*‐axis. Considering the linear relationship between the responding amplitude signals (*A*) calibrated through the *Q* factor and the drive voltage (*V*_tip_), the local piezoresponse can be obtained. As shown in Figure S14d (Supporting Information), the good linearity is the proof of the piezoelectric effect. The local piezoelectric coefficients of crystal *mono*‐I‐OA were evaluated to be 2.1 pC N^−1^, taking PVDF (28 pC N^−1^) as a benchmark. Notably, based on solvent selective effect, the newly formed *orth*‐I‐OA crystal shows enhanced piezoelectric performance. As its point group changes into *mm*2, the piezoelectric constant matrix [*d*] is shown below:

(2) $$\left[\begin{array}{cccccc} 0 & 0 & 0 & 0 & d_{15} & 0\\ 0 & 0 & 0 & d_{24} & 0 & 0\\ d_{31} & d_{31} & d_{33} & 0 & 0 & 0 \end{array}\right]$$

The piezoelectric tensor element *d*_33_ characterizes the vertical volume change as a response to an applied electric field in the vertical direction. At the tapping frequency of 110 Hz at room temperature, the piezoelectric coefficient of *orth*‐I‐OA is measured as 5 pC N^−1^, which is comparable to that of a typical ferroelectric croconic acid (5 pC N^−1^) (Figure 2c).^[^ ^37^ ^]^

**Figure 3:**
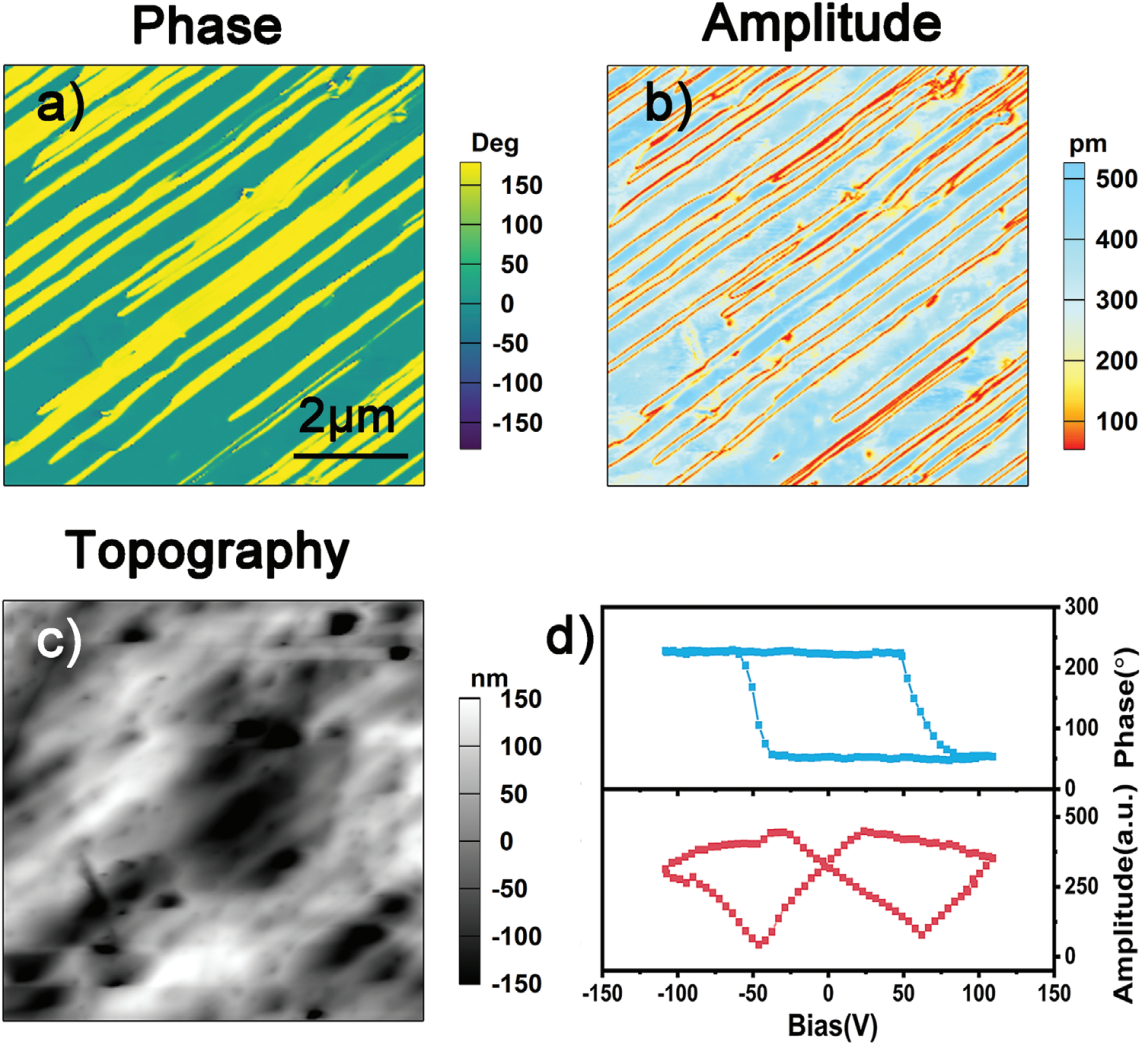
a) PFM phase, b) amplitude, and c) topography images for the single‐crystalline thin film of *orth*‐I‐OA. d) Amplitude and phase signals as functions of the tip voltage for a selected point, showing local PFM hysteresis loops.

*Mono*‐ and *orth*‐I‐OA crystallize in the polar point group 2 and *mm*2, which satisfies the essential condition of ferroelectricity. To determine their ferroelectricity, we used the PFM to nondestructively investigate the static domain structures as well as their dynamic behaviors at the nanoscale on the single‐crystalline thin films of *mono*‐ and *orth*‐I‐OA, respectively. Generally, PFM images contain two parts: the phase and amplitude image, which indicate the direction of polarization and the relative strength of the local piezoelectric coefficient in each domain, respectively. First, the vertical (out‐of‐plane) PFM images show an irregular domain structure with a clear phase contrast on the single‐crystalline thin film of *mono*‐I‐OA (Figure S14a–c, Supporting Information). Meanwhile, we clearly observed the stripe‐like domain pattern on the single‐crystalline thin film of *orth*‐I‐OA, resembling that of BiFeO_3_,^[^ ^38^ ^]^ which does not show obvious cross‐talk between the topography and domain structure (**Figure** **3**a–c). Then, we used the switching spectroscopy PFM (SS‐PFM) to understand the polarization switching behavior of *mono*‐ and *orth*‐I‐OA. SS‐PFM provides auxiliary proof for ferroelectricity through the PFM phase and amplitude response recorded by applying a sequence of DC voltages with a superimposed AC voltage to the PFM tip.^[^ ^39^, ^40^ ^]^ The vertical PFM phase and amplitude signals as a function of the DC bias appear as a hysteretic loop and a butterfly‐shaped curve (Figure 3d and Figure S14e,f, Supporting Information), which indicates the local hysteresis behavior of *mono*‐ and *orth*‐I‐OA.

**Figure 4:**
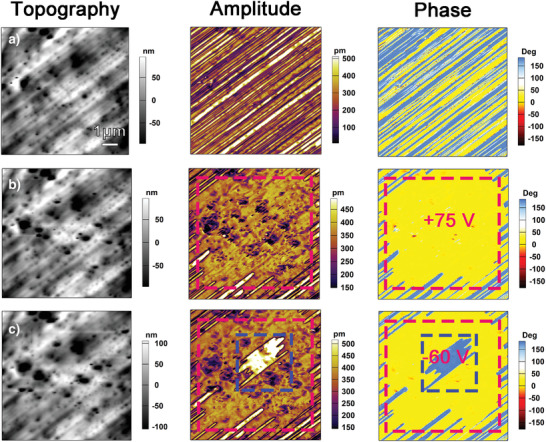
PFM images of *orth*‐I‐OA indicating ferroelectric polarization switching. Topography (left), amplitude (middle), and phase (right) images on the single‐crystalline thin film of *orth*‐I‐OA, recorded a) at the as‐grown state, after applying tip biases of b) +75 V, and c) subsequently −60 V on the central region.

For more visible and convincing evidence for ferroelectricity, the domain switching behavior on the single‐crystalline thin films of *mono*‐ and *orth*‐I‐OA should be taken into consideration. To determine the domain switching behavior of *orth*‐I‐OA, we applying of the DC bias of +75 V in the center region on a stripe‐like domain area, as shown in **Figure** **4**, the red marked rectangle region was switched into a uniform yellow domain. Then, a smaller center region (marked by a blue box) was written in the contact mode with an opposite tip voltage of −60 V, the written yellow domain was back switched by electric writing into a blue one. Consequently, a box‐in‐box domain pattern was formed observed from the amplitude and phase images shown in Figure 4, verifying the ferroelectric behavior of *orth*‐I‐OA. For *mono*‐I‐OA, as shown in Figure S15 (Supporting Information), the similar box‐in‐box domain pattern could be obtained from the amplitude and phase images after the applying of the DC bias of +60 V in the center region (marked by a green box) and then an opposite tip voltage of −50 V for 1 s in the center (marked by a pink box). These electrically writable box‐in‐box patterns found on the single‐crystalline thin film of *mono*‐ and *orth*‐I‐OA provide solid evidence for their ferroelectricity.

We then carried out polarization–electric field (*P–E*) hysteresis loop measurements to confirm the intrinsic ferroelectric properties on the single‐crystalline thin film of *orth*‐I‐OA using the double‐wave method at room temperature. As shown in Figure 2d, there are two opposite peaks in the *J–V* curve, which indicates two stable states with opposite polarization which could be obtained through polarization switching. ^[^ ^41^, ^42^ ^]^ A typical *P–E* hysteresis loop can be obtained according to the current accumulating and the measured *P*_s_ of *orth*‐I‐OA is about 1.55 µC cm^−2^. Due to the polymorphism caused by the solvent selective effect, we calculated the vector sum of the dipole moment of the I‐OA molecule in the corresponding unit cell to estimate the ferroelectric polarization of the *mono*‐ and *orth*‐I‐OA crystals. As shown in Figure S16 (Supporting Information), the dipole moment of the I‐OA molecule is about 2.931 Debye. For *mono*‐I‐OA, the estimated *P*_s_ value is about 0.04 µC cm^−2^ (along the *b*‐axis), which is relatively small. While for *orth*‐I‐OA, if all molecular dipoles contribute 100% to the total polarization, the *P*_s_ should be 3.90 µC cm^−2^. In fact, there is an obvious misalignment between the direction of the molecular dipole moment and the crystallographic polar *c*‐axis. In order to gain a more accurate value of ferroelectric polarization, we employed the Berry phase method and the estimated *P*_s_ value of 3.43 µC cm^−2^ (along the *c*‐axis) can be obtained for *orth*‐I‐OA. We thus estimated the total *P*_s_ value to be about 3.43 µC cm^−2^ for *orth*‐I‐OA. This calculated *P*_s_ value is larger than those of some ferroelectrics like 2,2,6,6‐tetramethylpiperidine‐1‐oxyl (0.5 µC cm^−2^) and trichloroacetamide (0.2 µC cm^−2^).^[^ ^43^, ^44^ ^]^ Consequently, we are delighted to find that the solvent selective effect could not only enhance the SHG intensity and piezoelectric response *d*_33_ but also the *P_s_* value of I‐OA, and it would be a satisfying method for the optimization of ferroelectric and piezoelectric performance.

**Figure 5:**
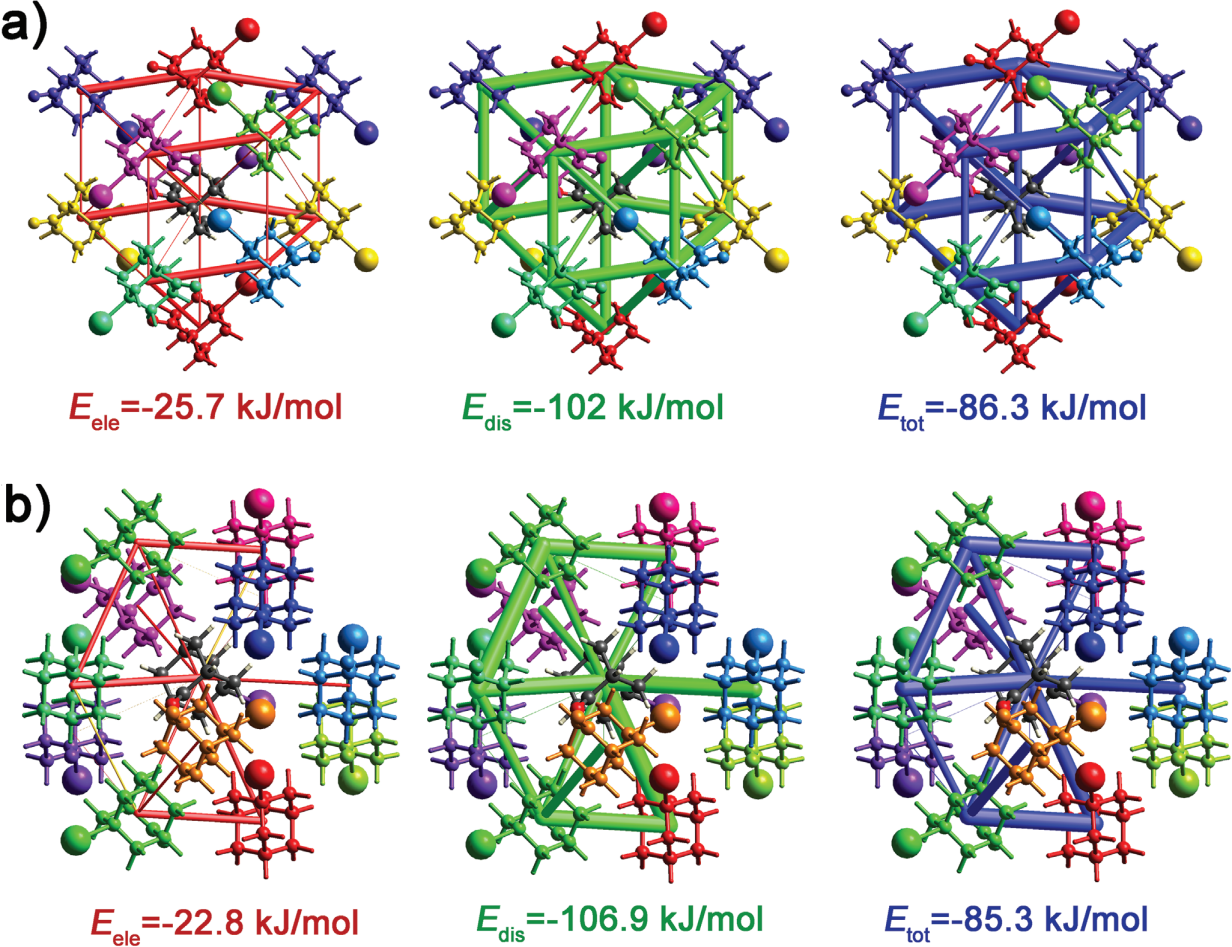
Energy frameworks corresponding to different energy components and total interaction energy of a) the *orth*‐I‐OA and b) the *mono*‐I‐OA forms. Red, green, and blue color codes represent electrostatic (*E*_ele_), dispersion (*E*_dis_), and the total (*E*_tot_) interaction energies, respectively.

To investigate the stability of the two crystal phases induced by the solvent selective effect, we performed the interaction energy calculations using the program *CrystalExplorer*17.5 (details are shown in Supporting Information) and the UNI force field as implemented in the program Mercury. As depicted in Figure S17 (Supporting Information), targeting at the selected central molecule in *mono*‐ and *orth*‐I‐OA crystal, the neighboring molecules (default radius of 3.8 Å) which have interactions with it are marked with different colors and their energies between different pairs are calculated. The energy frameworks of *mono*‐ and *orth*‐I‐OA form triangular and triangular/quadrilateral frameworks, respectively, where the thickness of the rods indicate the relative intensity of interaction energy. **Figure** **5** shows the energy frameworks of *orth*‐I‐OA and *mono*‐I‐OA observed along the *c*‐axis. The *mono*‐form has slightly higher electrostatic interaction energy, and the *orth*‐form shows higher dispersion energy. Meanwhile, the total energies of *mono*‐ and *orth*‐I‐OA are calculated as −85.3 and −86.3 kJ mol^−1^ according to the interaction energies calculation, respectively. Furthermore, the intermolecular interaction energies have also been calculated based on the UNI force field in Mercury, which is similar to that of the energy framework analysis. *Orth*‐I‐OA has slightly lower interaction energy (−90.1 kJ mol^−1^) than that of the *mono*‐I‐OA form (−88.6 kJ mol^−1^). All in all, the similar interaction energies of *mono*‐ and *orth*‐I‐OA explain the reason why they have similar melting points. Most importantly, the close interaction energies make the transformation between the two crystal phases by changing the solvent easy, and also conclude that the thermodynamic effect may not be responsible for the selective behavior of a solvent. Thus, in good accordance with the above‐mentioned speculations, we favor the kinetic mechanism to explain this solvent selective effect realized in I‐OA, where the solvent shows selective adsorption to some certain faces, and thereby changes the crystal phases as well as their physical performance.^[^ ^45^ ^]^

## 3 Conclusions

In summary, based on the nonferroelectric 2‐adamantanone, we have adopted the chemical design strategy of lowering molecular symmetry to successfully design an organic ferroelectric I‐OA. Accompanied by the introducing of halogen atoms on the OA molecule from F to I, the crystal symmetries transform gradually from a plastic cubic phase into a polar space group *P*2_1_ (*mono*‐I‐OA) at room temperature possessing ferroelectricity but poor piezoelectric and ferroelectric performance. Based on solvent selective effect, we obtained another polymorphic crystal *orth*‐I‐OA, which crystallized in the orthorhombic polar space group *Pna*2_1_ at room temperature, which shows a larger *P*_s_ value of 3.43 µC cm^−2^, stronger SHG intensity and greater piezoelectric response of 5 pC N^−1^ in comparison with its *mono*‐phase. This inducing of polymorphism could be attributed to the polarity of solvents. This paves a way for the chemical design of single‐component ferroelectrics as well as their performance optimization.

## 4 Experimental Section

### Synthesis

5‐Fluoro‐2‐adamantanone (F‐OA):^[^ ^46^ ^]^ 5 g (21 mmol) of 5‐bromo‐2‐adamantanone (Br‐OA) and 8 g (63 mmol) AgF were refluxed in 150 mL of dry cyclohexane for 12 h. The product was retrieved with ether. The combined organic fractions were washed neutral with NaHCO_3_, brine, and water, and dried over Na_2_SO_4_. Removal of the solvent in vacuo and then white solid of 5‐fluoro‐2‐adamantanone was obtained. ^1^H NMR (300 MHz) *δ* 2.69 (d, *J* = 4.5 Hz, 2H), 2.44 (q, *J* = 3.3 Hz, 1H), 2.30–2.18 (m, 2H), 2.13 (dtd, *J* = 8.6, 4.6, 4.2, 2.3 Hz, 4H), 1.97 (q, *J* = 3.0, 2.5 Hz, 4H) (Figure S18, Supporting Information). ^19^F NMR (282 MHz) *δ* ‐140.27 (dq, *J* = 7.9, 4.6, 3.8 Hz) (Figure S19, Supporting Information). GC‐MS calcd. for C_10_H_13_FO 168, found 168.10.

5‐Chloro‐2‐adamantanone (Cl‐OA):^[^ ^47^ ^]^ 12 mL of thionyl chloride were added to 2.0 g (12 mmol) of 5‐hydroxy‐2‐adamantanone and refluxed for 2 h. Afterward, the thionyl chloride was removed into vacuo and the residue was dissolved in CH_2_Cl_2_. The organic was washed two times with NaOH (0.24 g) solution, two times with 10% NaCl solution, and dried over anhydrous Na_2_SO_4_. Removal of the solvent in vacuo and then white solid of 5‐chloro‐2‐adamantanone was obtained. ^1^H NMR (300 MHz) *δ* 2.64 (s, 2H), 2.52–2.24 (m, 7H), 2.11–1.90 (m, 4H) (Figure S20, Supporting Information). GC‐MS calcd. for C_10_H_13_ClO 184, found 184.65.

5‐Bromo‐2‐adamantanone (Br‐OA):^[^ ^47^ ^]^ 2.5 g (15 mmol) of 5‐hydroxy‐2‐adamantanone was dissolved in 30 mL of 40% HBr solution and refluxed for 7 h. After the addition of 25 mL of water, the mixture was extracted three times with diethyl ether. The organic phase was washed with 10% NaCl solution and dried over anhydrous Na_2_SO_4_. The yellow solid of 5‐bromo‐2‐adamantanone was obtained. ^1^H NMR (300 MHz) *δ* 2.72–2.45 (m, 8H), 2.26 (q, *J* = 3.4 Hz, 1H), 2.16–1.92 (m, 4H) (Figure S21, Supporting Information). GC‐MS calcd. for C_10_H_13_BrO 228, found 229.10.

5‐Iodo‐2‐adamantanone (I‐OA):^[^ ^48^ ^]^ 2.5 g (15 mmol) of 5‐hydroxy‐2‐adamantanone was added to 3.85 mL of hexane and 15 mL of benzene, then 10.5 mL 55% freshly distilled HI solution was added in, the mixture was refluxed for 24 h. After the mixture was cooled to room temperature, additional 10 mL benzene was added in and then removed the aqueous layer. The benzene layer was washed with water, Na_2_S_2_O_3_ and NaHCO_3_ solutions and then dried, finally, the solvent was removed. The yellow solid of 5‐iodo‐2‐adamantanone was obtained. ^1^H NMR (300 MHz) *δ* 2.97–2.66 (m, 6H), 2.49 (q, *J* = 2.5 Hz, 2H), 2.24–1.99 (m, 5H) (Figure S22, Supporting Information). GC‐MS calcd. for C_10_H_13_IO 276, found 276.10.

### Crystal Growth

F‐OA, Cl‐OA, Br‐OA, and I‐OA were recrystallized using the method of slow evaporation of the solution. F‐OA was dissolved in ether, Cl‐OA and Br‐OA were respectively dissolved in ethyl acetate. The clear solutions were put to evaporate at room temperature for 3 d. Then the white and pale‐yellow bulk crystals were obtained, respectively. I‐OA was dissolved in 8 solvents including petroleum ether, cyclohexane, toluene, ether, ethyl acetate, dichloromethane, acetone, and acetonitrile to obtain the yellow plate *mono*‐I‐OA crystals. *Orth*‐I‐OA crystal was obtained as pale‐yellow needle crystal in 4 solvents including ethanol, methanol, DMF, and DMSO.

### Preparation of Thin Film and PFM Measurement

The single‐crystalline thin films of *mono*‐ and *orth*‐I‐OA were used for PFM measurement. First, the powders of *mono*‐ and *orth*‐I‐OA were dissolved in ethyl acetate and ethanol to obtain the precursor solutions with a concentration of 75 mg mL^‐1^, respectively. Then, 20 µL of their corresponding precursor solution was carefully dropped on the cleaned ITO (indium tin oxide)‐coated glass. For the PFM and *P*–*E* hysteresis loop measurement, the thickness of the as‐grown single‐crystalline thin films of *mono*‐ and *orth*‐I‐OA are about 1.5 and 2.2 µm, respectively. Domain imaging, PFM spectroscopy, and polarization switching studies were carried out on the single‐crystalline thin film of *mono*‐ and *orth*‐I‐OA through the conductive Pt/Ir‐coated silicon probes (EFM, Nanoworld) at 298 K. The resonant‐enhanced PFM mode was used to enhance the signal, with a typical AC voltage frequency of ≈320 kHz and an AC amplitude of 2.0 V.

### Differential Scanning Calorimetry (DSC), Dielectric Measurements, Second Harmonic Generation (SHG) Measurement, and Thermogravimetric Analysis (TGA) Measurement

The powder samples of OA, Cl‐OA, Br‐OA, *mono*‐ and *orth*‐I‐OA were encased in the aluminum crucibles to perform the DSC measurements on a PerkinElmer Diamond DSC instrument under the nitrogen atmosphere. The complex permittivity was measured on Tonghui TH2828A. Silver conduction paste deposited on the plate surfaces was used as the electrodes. SHG measurement was performed by using the instrument model of Ins 1210058, INSTEC Instruments on the powder samples of OA, Cl‐OA, Br‐OA, *mono*‐ and *orth*‐I‐OA, and the laser is Vibrant 355 II, OPOTEK at a wavelength of 1064 nm, 5 ns pulse duration, 1.6 MW peak power, 10 Hz repetition rate. TGA measurement was performed on PerkinElmer TGA 8000 from 300 K to 900 K at the rate of 30 K min^‐1^.

### X‐ray Crystallographic, Powder X‐ray Diffraction (PXRD), Infrared (IR), ^1^H Nuclear Magnetic Resonance Spectroscopy Measurements

The single‐crystal X‐ray diffraction data of Cl‐OA, Br‐OA, *mono*‐I‐OA and *orth*‐I‐OA were collected at 298 K on the Rigaku Oxford diffractometer (Cu K*α*, *λ* = 1.54184 Å). The structures were determined and refined by the direct methods with the full‐matrix least‐squares method based on F2 in the SHELXTL program package. All the non‐H atoms were anisotropically refined using all reflections with *I* > *2σ*(*I*). The asymmetric unit and packing images were drawn by the DIAMOND. Variable‐temperature PXRD measurements were performed on a Rigaku D/MAX 2000 PC X‐ray diffractometer in the 2*θ* range of 5 to 50° with a step size of 0.02°. The infrared spectra were obtained on a Fourier Transform Infrared spectrometer (EQUINOX 55, Bruker, Germany). The samples were prepared by grinding the dried powder of *mono*‐ and *orth*‐I‐OA with KBr together, and then compressed into thin pellets. The ^1^H nuclear magnetic resonance spectroscopy measurement was performed on the Bruker AVANCE NEO 300 MHz NMR spectrometer with 2 mg of the samples dissolved in 0.75 mL deuterated chloroform solvent.

### Hirshfeld Surfaces, Fingerprint Plots, and the Interaction Energy Calculation

Molecular Hirshfeld surface calculations were performed by using the *CrystalExplorer*17.5 program. All bond lengths to hydrogen were automatically modified to typical standard neutron values. In this study, all the Hirshfeld surfaces were generated using a standard (high) surface resolution. The intensity of molecular interaction is mapped onto the Hirshfeld surface by using a red‐blue‐white color scheme: where the white regions exactly correspond to the distance of Van der Waals contact, the blue ones correspond to longer contacts, and the red ones represent closer contacts. The interaction energy calculation was using the *CrystalExplorer*17.5 program with the energy model of HF/3‐21G and the UNI force field as implemented in the program Mercury with the hydrogens all being normalized .

### Experimental Details of Calculation

The dipole moment was calculated at b3lyp/6‐31G(d) level (SDD for I) with Gaussian 16 software. Molecular conformation based on the experimentally measured single‐crystal X‐ray diffraction structure was constructed. Density functional calculations based on the Berry phase method developed by Kingsmith and Vanderbilt were carried out. The first‐principles calculations were performed within the framework of density functional theory implemented in the Vienna ab initio Simulation Package (VASP; 5.4.4). The energy cutoff for the expansion of the wave functions was fixed to 550 eV and the exchange−correlation interactions were treated within the generalized gradient approximation of the Perdew−Burke−Ernzerhof type. First, the geometrical optimization was performed by fixing the lattice constant based on the experimentally X‐ray crystal structure. Then, the Berry phase calculation was calculated on the optimized geometry.

[CCDC 2161120, 2133253 and 2152825–2152827 contains the supplementary crystallographic data for this paper. These data can be obtained free of charge from The Cambridge Crystallographic Data Centre via www.ccdc.cam.ac.uk/data_request/cif.]

## Conflict of Interest

The authors declare no conflict of interest.

## Supporting information

Supporting Information

## Data Availability

Research data are not shared.
