# Supplementary material for: Solvent Selective Effect Occurs in Iodinated Adamantanone Ferroelectrics
Source: Adv Sci (Weinh). 2022 Apr 25;9(18):2201702. doi: 10.1002/advs.202201702 (PMC9218660; doi:10.1002/advs.202201702)
Supplement: Supplementary file 1 — Supporting Information [file ADVS-9-2201702-s001.pdf]

ADVANCED SCIENCE
Open Access

Supporting Information

for *Adv. Sci.*, DOI 10.1002/advs.202201702

Solvent Selective Effect Occurs in Iodinated Adamantanone Ferroelectrics

*Lei Xu, Yao Zhang, Huan-Huan Jiang, Nan Zhang, Ren-Gen Xiong and Han-Yue Zhang**

Supporting Information

Solvent Selective Effect Occurs in Iodinated Adamantanone Ferroelectrics

*Lei Xu, Yao Zhang, Huan-Huan Jiang, Nan Zhang, Ren-Gen Xiong, Han-Yue Zhang**

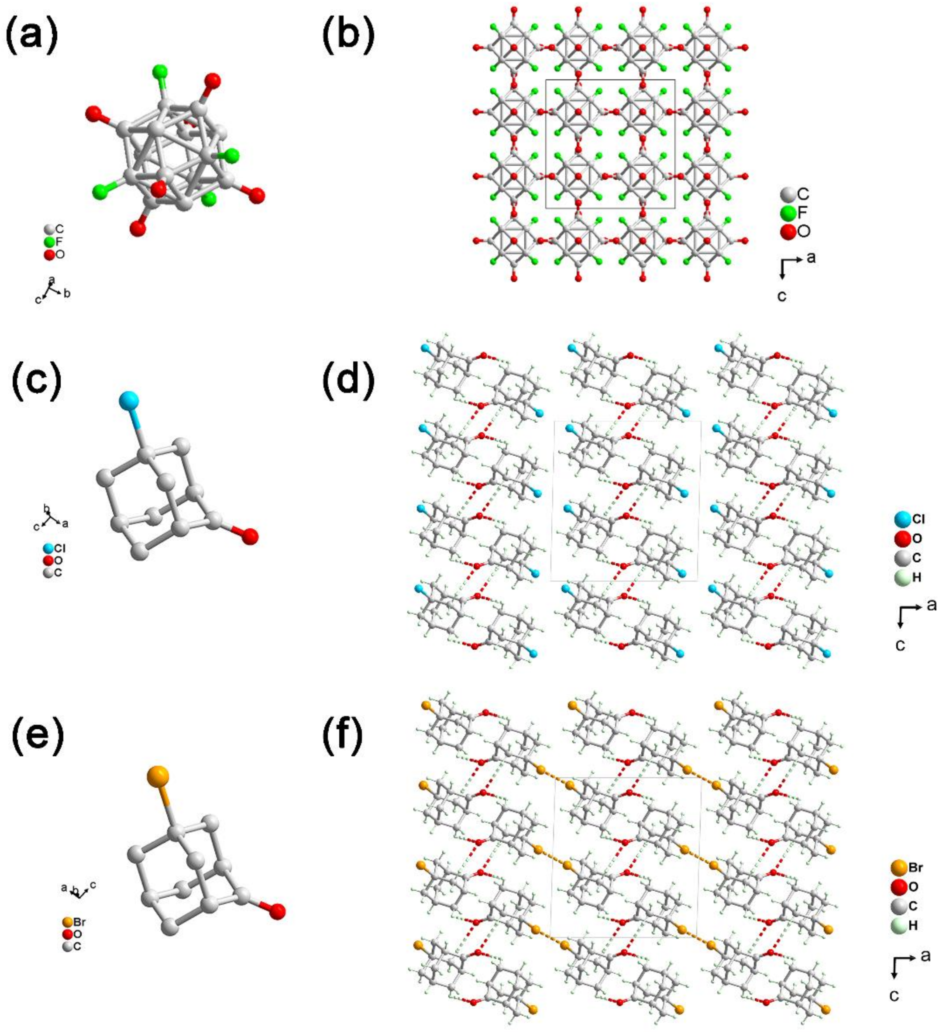

Figure S1. The spherical disorder conformation of 5-fluoro-2-adamantanone (F-OA) (a). Packing view of F-OA along the *b*-axis at 293 K (b). The basic unit of compound 5-chloro-2-adamantanone (Cl-OA) (c) and 5-bromo-2-adamantanone (Br-OA) (e). Packing views of Cl-OA (d) and Br-OA (f) along the *b*-axis at 298 K, respectively. The red dashed lines in both (d) and (f) refer to the weak C–H···O–C molecular interactions of Cl-OA and Br-OA, and the yellow dashed lines in (f) refer to the weak halogen···halogen interaction between the Br atoms of Br-OA.

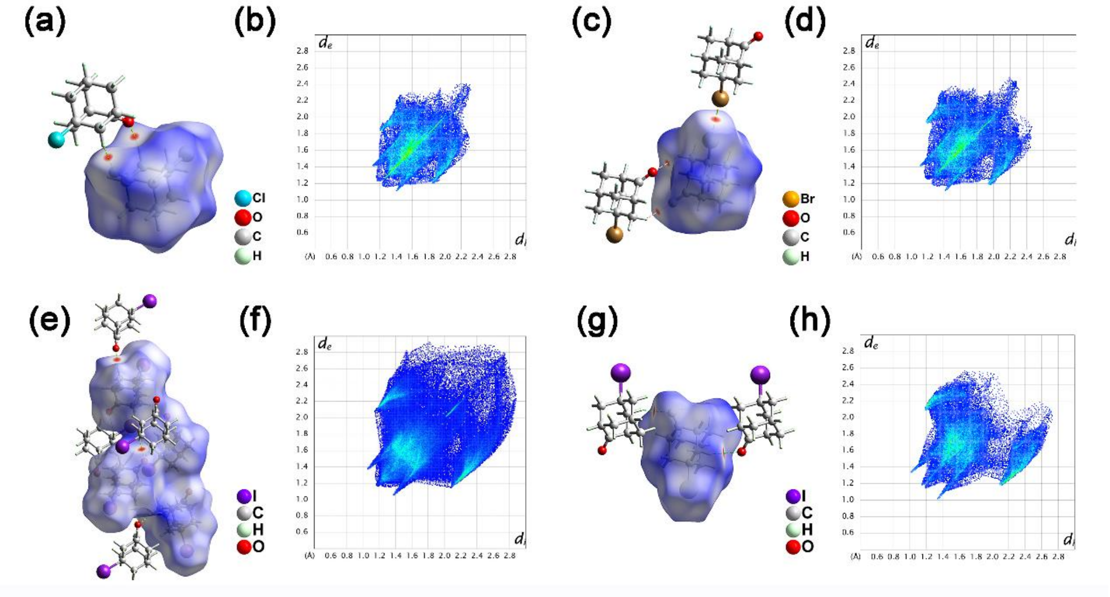

Figure S2. The Hirshfeld d_{norm} surfaces and the 2D fingerprint plots of compound Cl-I-OA, Br-I-OA, *mono*-5-iodo-2-adamantanone (*mono*-I-OA) and *orth*-5-iodo-2-adamantanone (*orth*-I-OA).

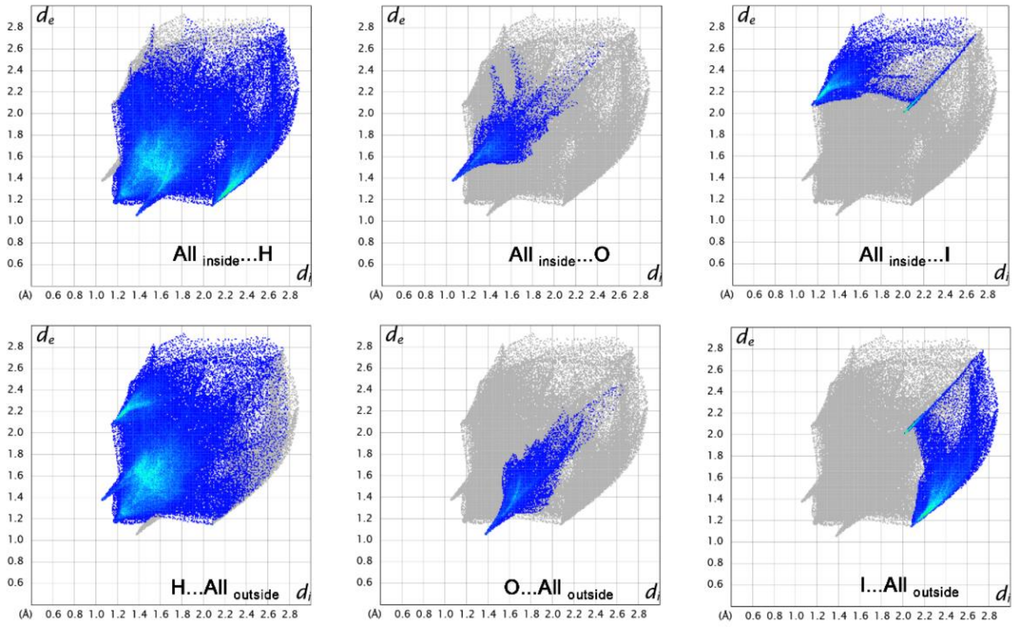

Figure S3. The 2D fingerprint plots of *mono*-I-OA. The interactions between different atoms are marked in the Figures.

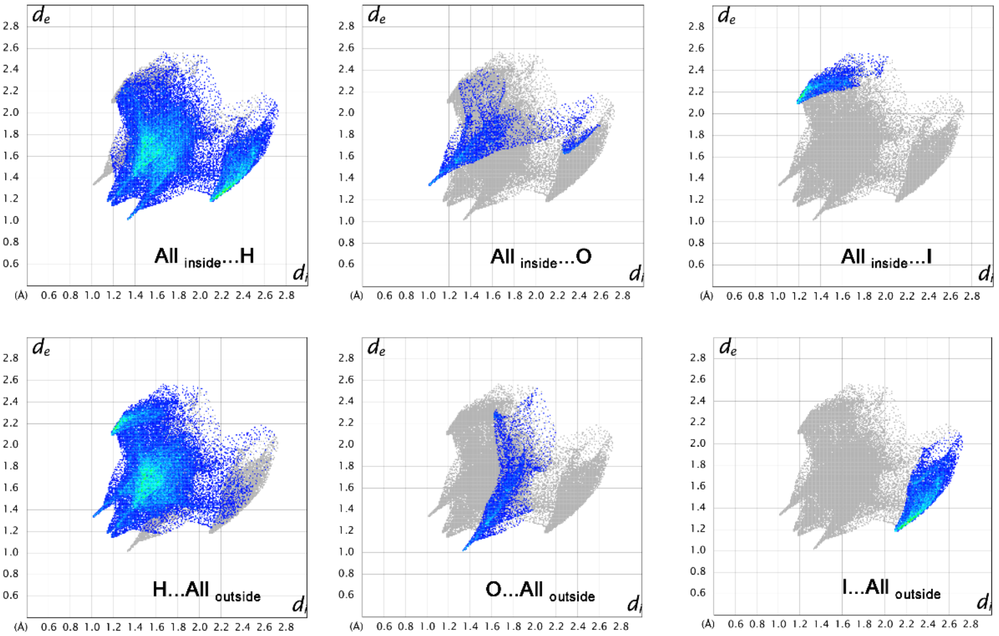

Figure S4. The 2D fingerprint plots of *orth*-I-OA. The interactions between different atoms are marked in the Figures.

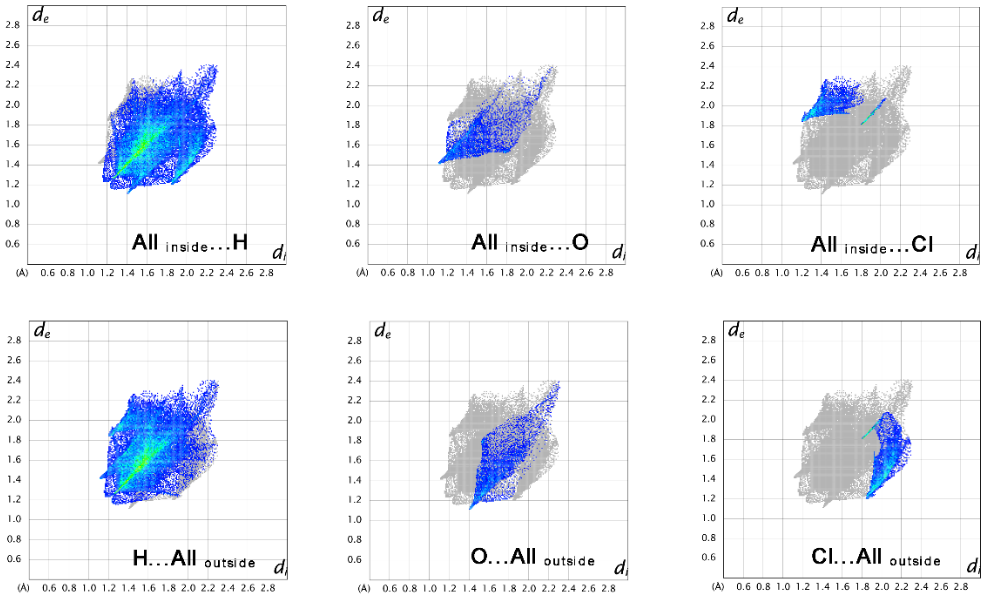

Figure S5. The 2D fingerprint plots of Cl-OA. The interactions between different atoms are marked in the Figures.

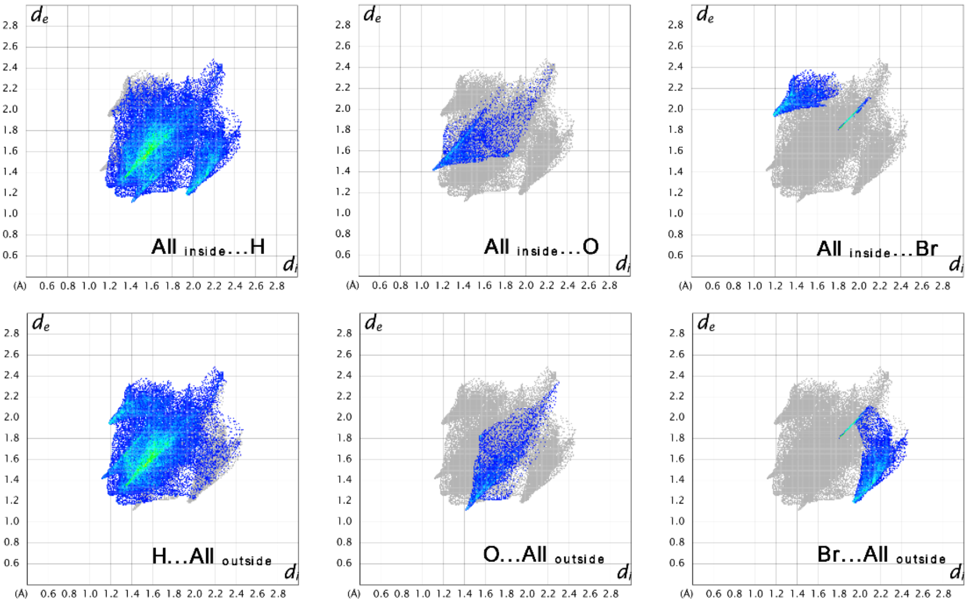

Figure S6. The 2D fingerprint plots of Br-OA. The interactions between different atoms are marked in the Figures.

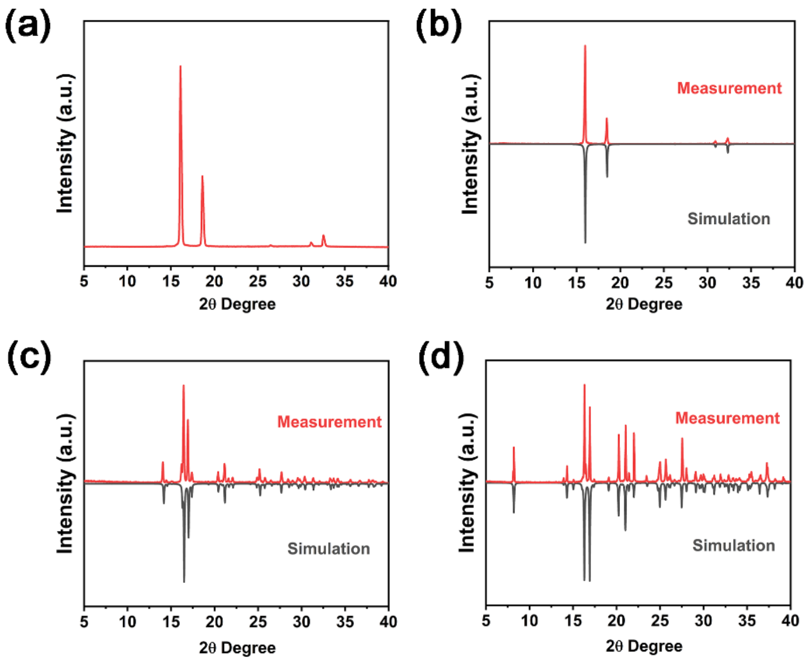

Figure S7. PXRD patterns of 2-adamantanone (OA) (a) at 298 K, F-OA (b) at 293 K, Cl-OA (c) at 298 K, Br-OA (d) at 298 K, the red line and the black line represent measured and simulated patterns, respectively.

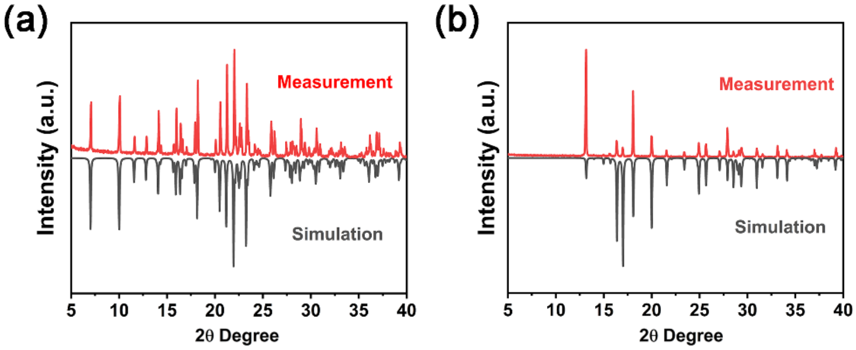

Figure S8. PXRD patterns of *mono*-I-OA (a) and *orth*-I-OA (b) at 298 K, the red line and the black line represent measured and simulated patterns, respectively.

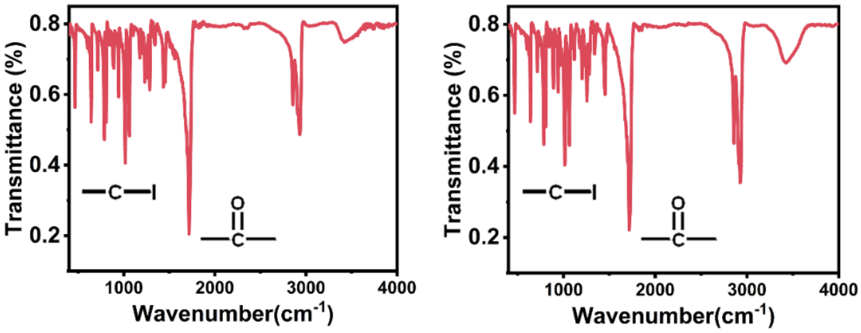

Figure S9. The IR spectra of *mono*-I-OA and *orth*-I-OA at 298 K.

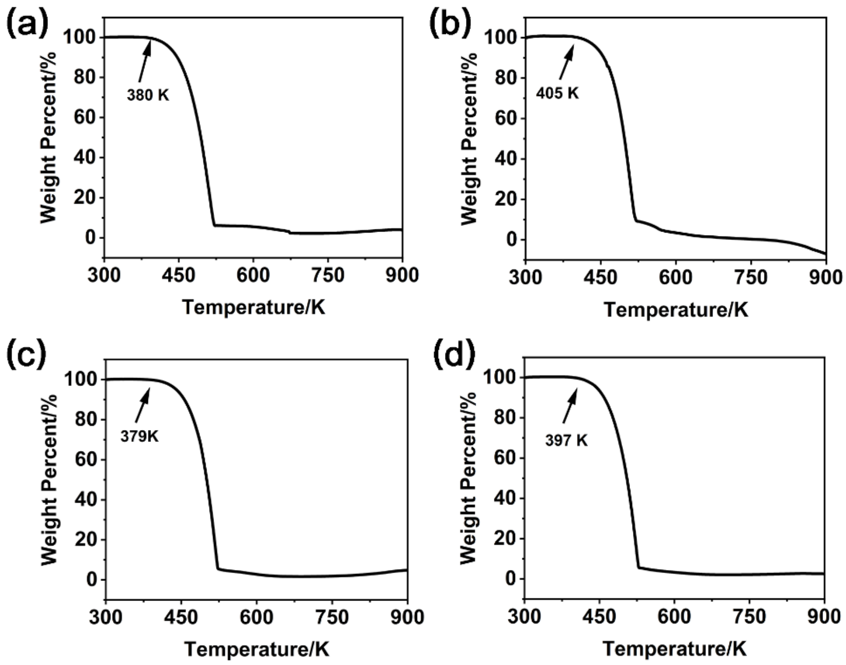

Figure S10. TGA curves of compound OA (a), F-OA (b), Cl-OA (c), Br-OA(d) in the temperature range of 300 K to 900 K.

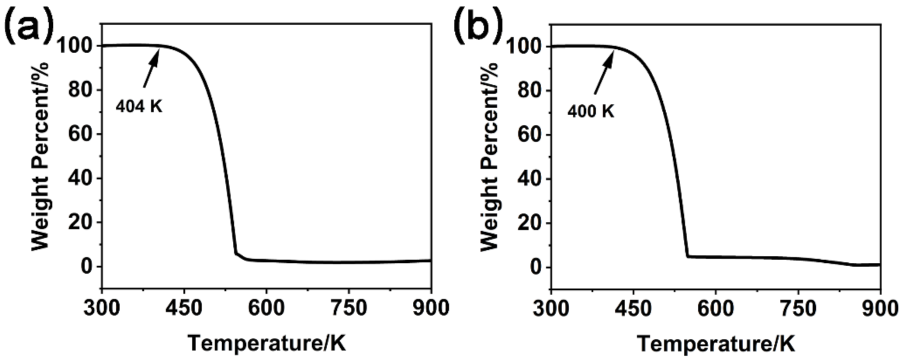

Figure S11. TGA curves of compound *mono*-I-OA (a) and *orth*-I-OA (b) in the temperature range of 300 K to 900 K.

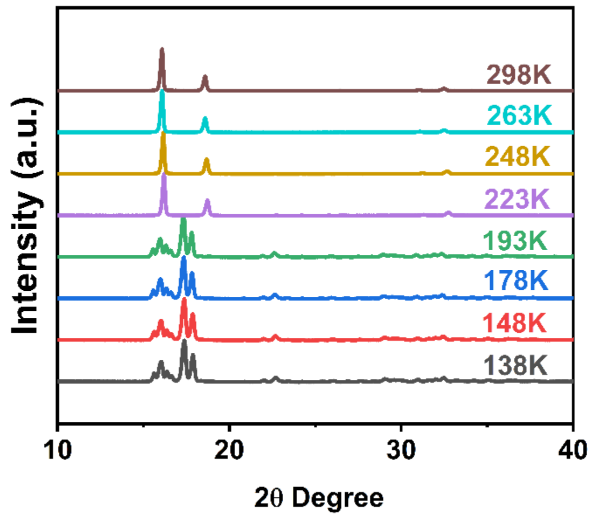

Figure S12. Variable-temperature PXRD patterns of OA.

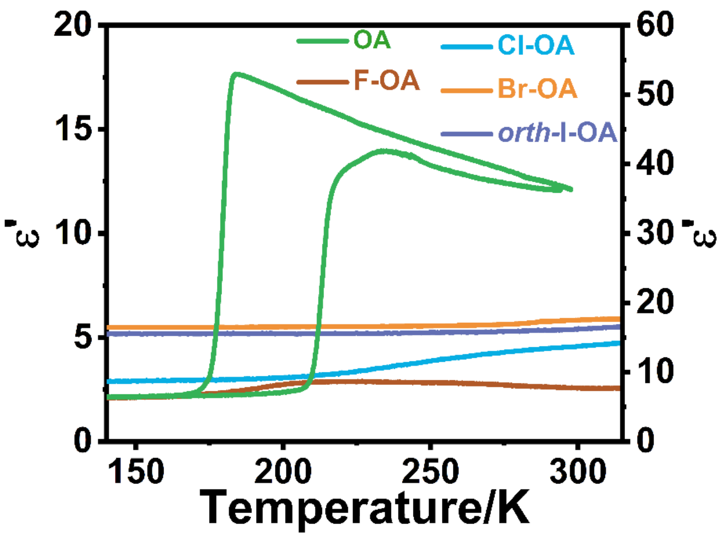

Figure S13. Temperature-dependent ε' at 1 MHz in the heating-cooling cycles of OA, F-OA, Cl-OA, Br-OA and *orth*-I-OA.

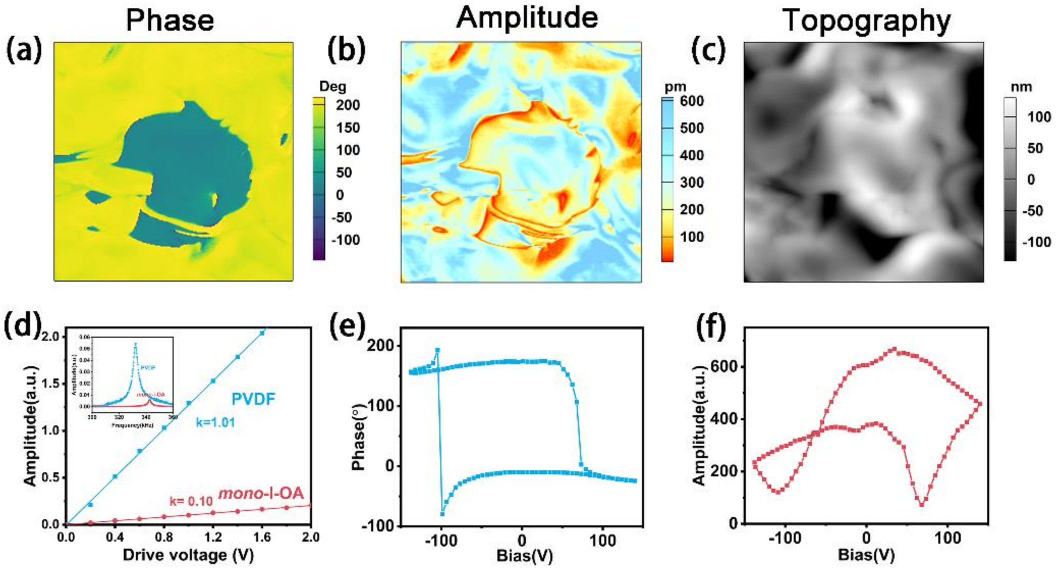

Figure S14. PFM phase (a), amplitude (b), and topography (c) images for the thin film of *mono*-I-OA. (d) Comparison of PFM resonance peaks (insert) and effective piezoelectric coefficient of the films of PVDF and *mono*-I-OA. Phase (e) and amplitude (f) signals as functions of the tip voltage for a selected point, showing local PFM hysteresis loops.

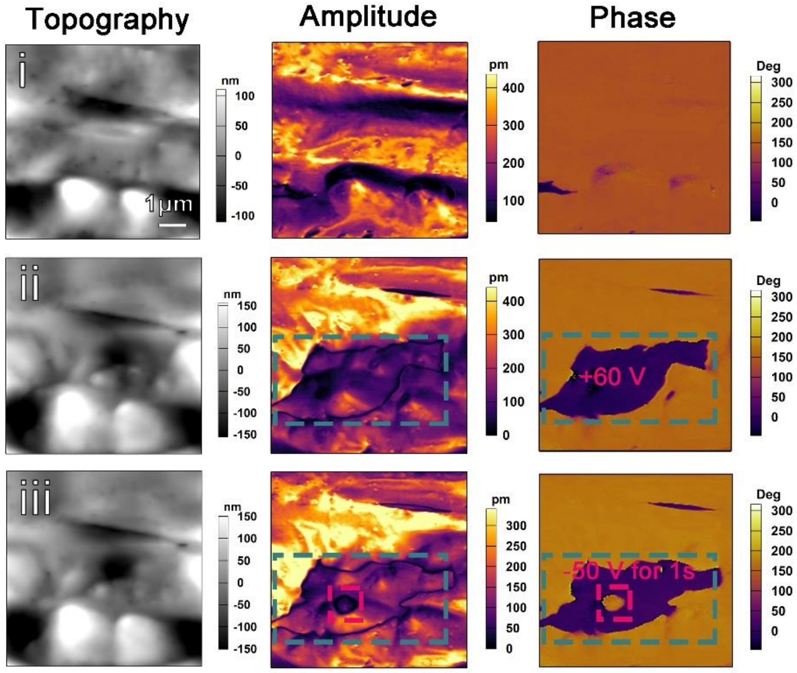

Figure S15. PFM images of *mono*-I-OA indicating ferroelectric polarization switching. Topography (left), amplitude (middle), and phase (right) images on the thin film of *mono*-I-OA, recorded (i) at the as-grown state, after applying tip biases of (ii) +60 V, and (iii) subsequently –50V for 1s on the central region.

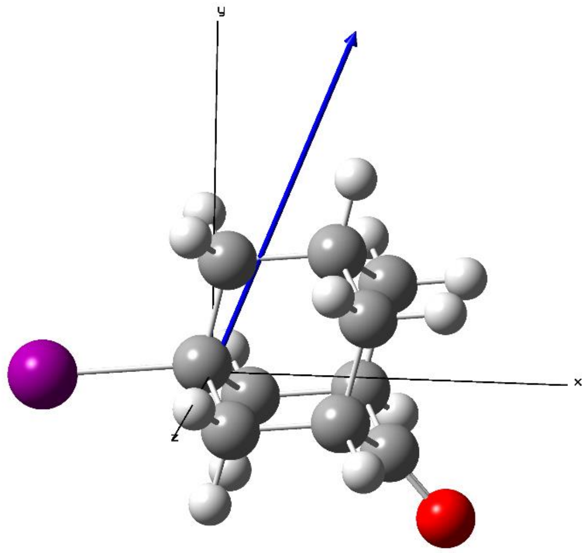

Figure S16. Molecular structure of *orth*-I-OA with 2.931 Debye, the vector components of the molecular dipole on the x-, y- and z axis are 1.076, 2.726, and 0 Debye, respectively.

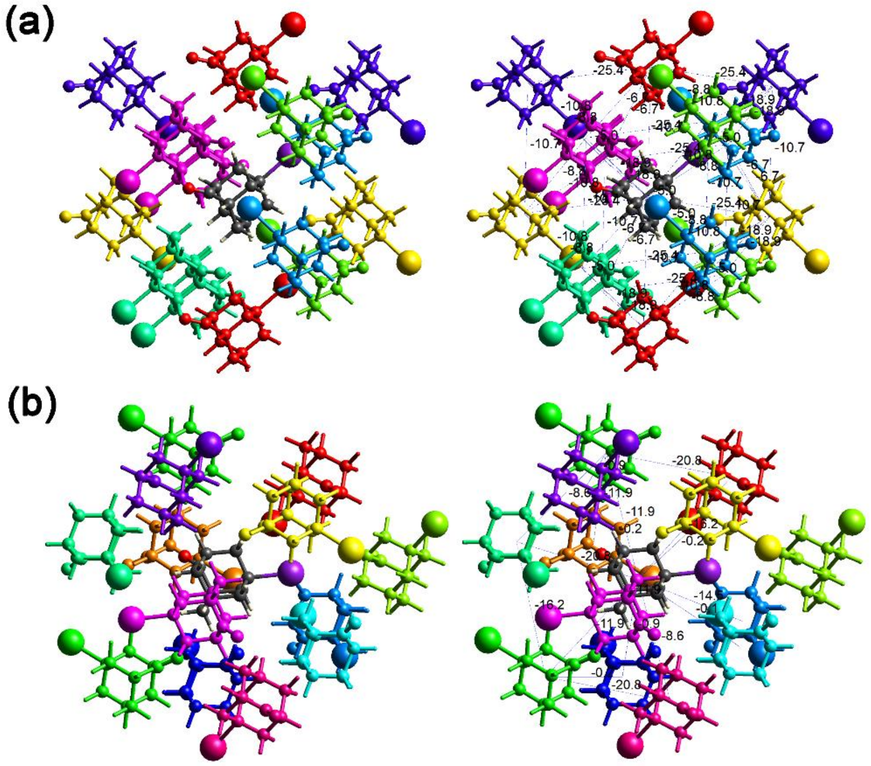

Figure S17. The left figures are color coding for the neighboring molecules around molecules and the right figures are energies between different pairs of *orth*-I-OA (a) and *mono*-I-OA (b). The central molecules are shown with atom type color.

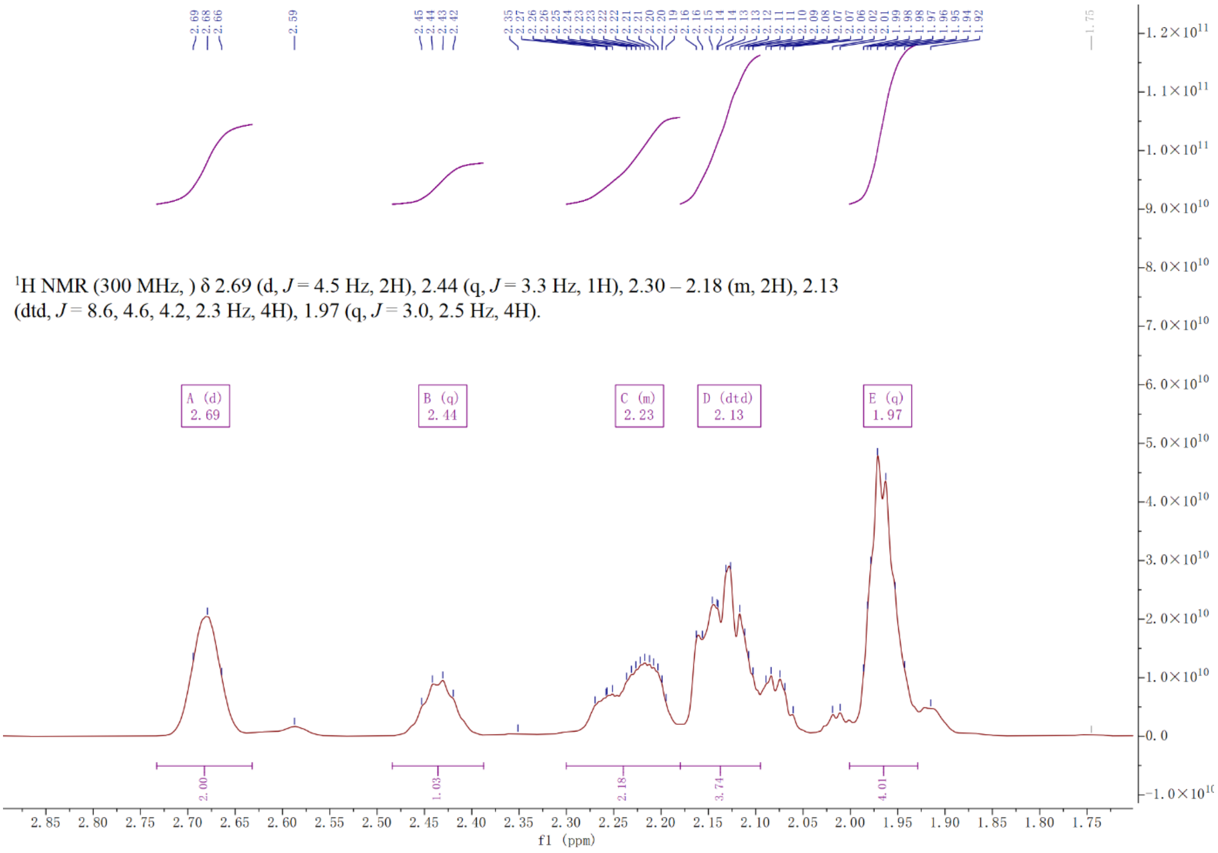

Figure S18. The ^{1}H NMR (300 MHz) spectrum of F-OA.

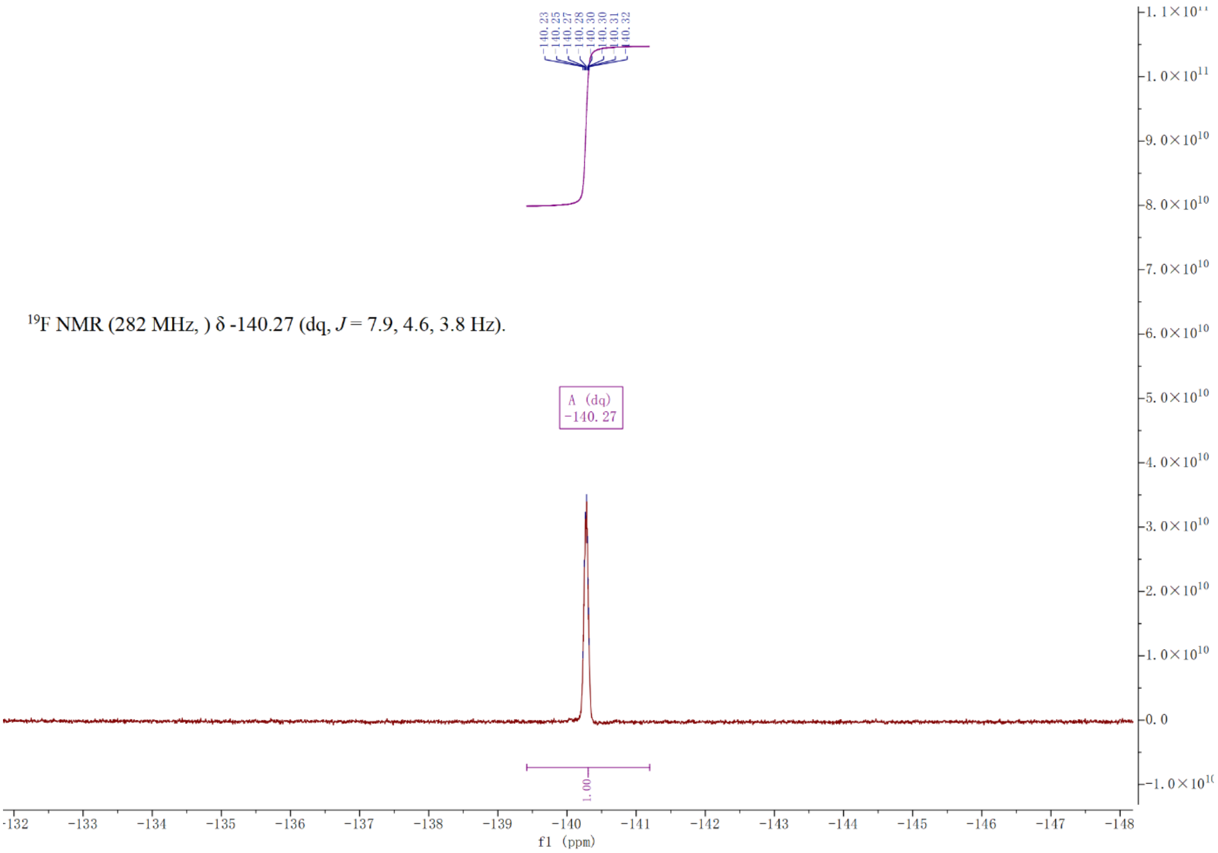

Figure S19. The ^{19}F NMR (282 MHz) spectrum of F-OA.

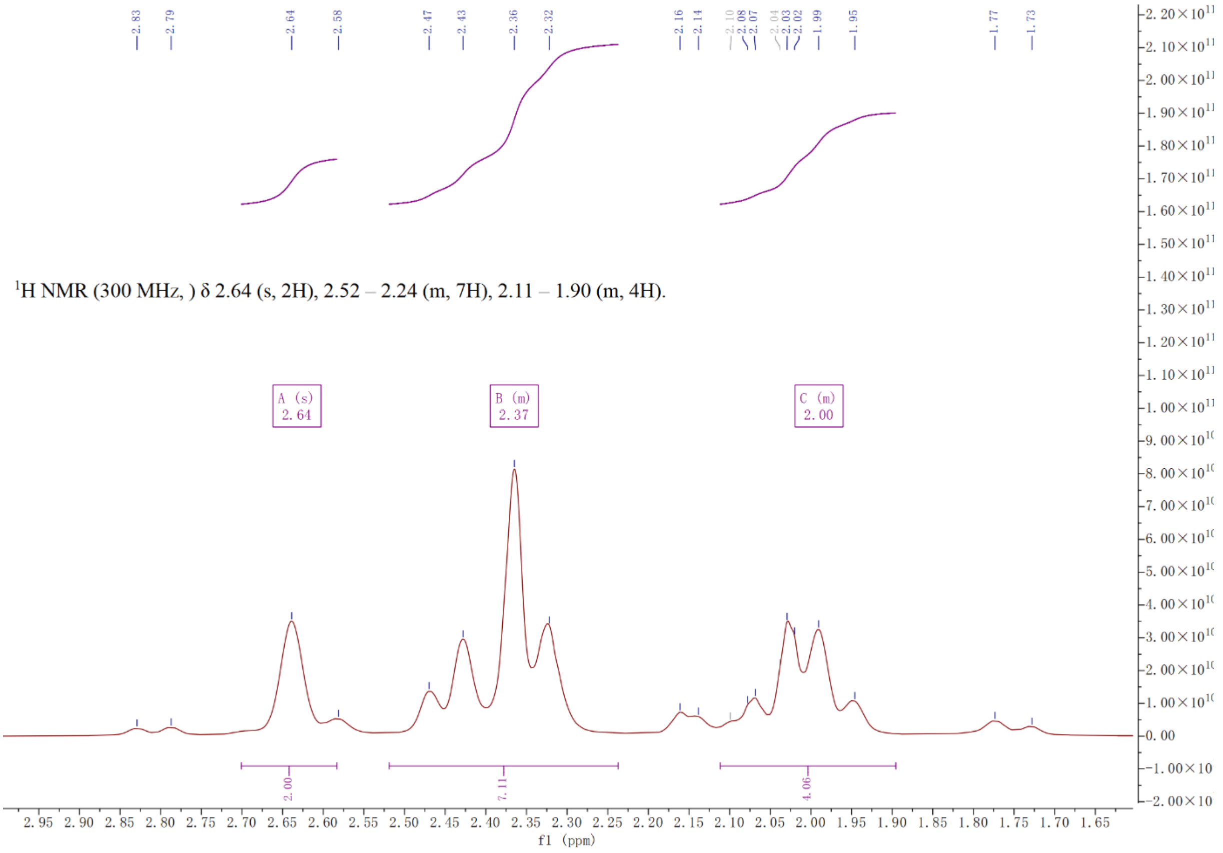

Figure S20. The ^{1}H NMR (300 MHz) spectrum of Cl-OA.

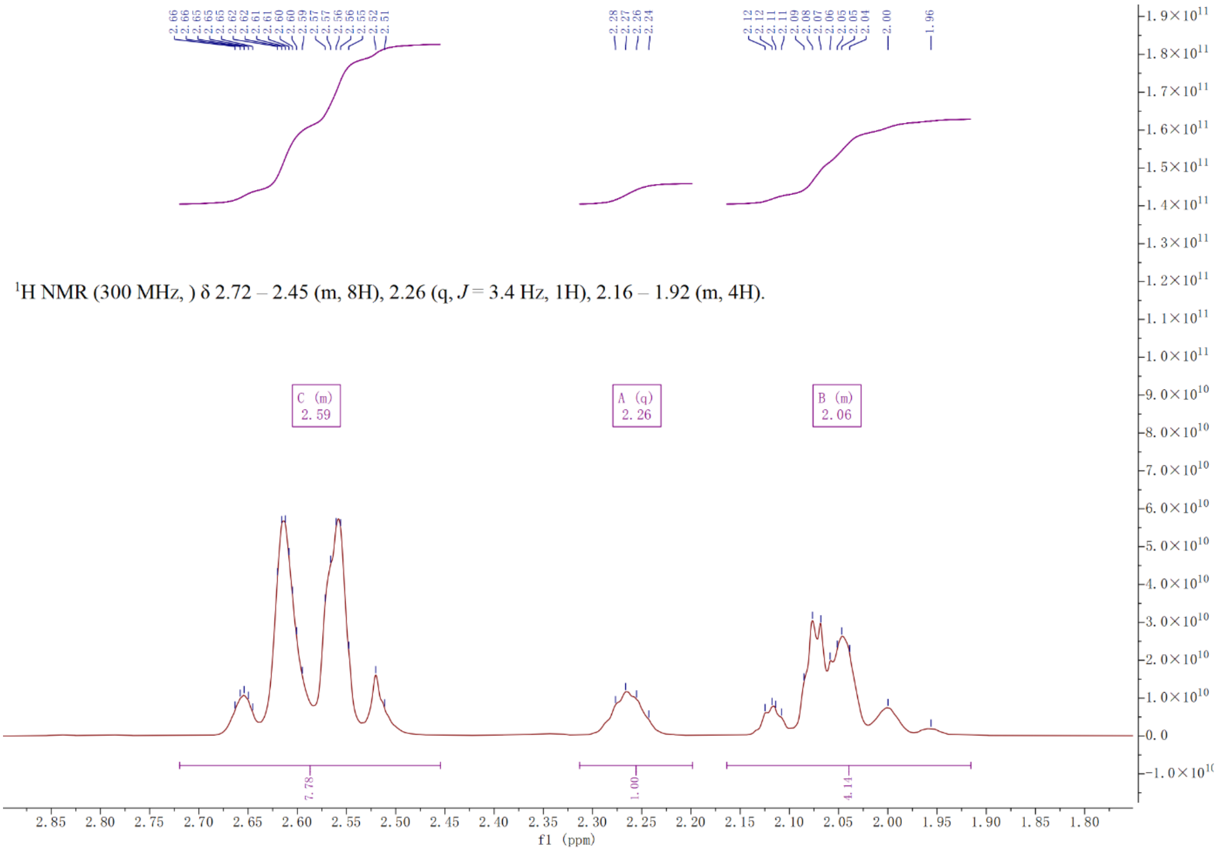

Figure S21. The ^{1}H NMR (300 MHz) spectrum of Br-OA.

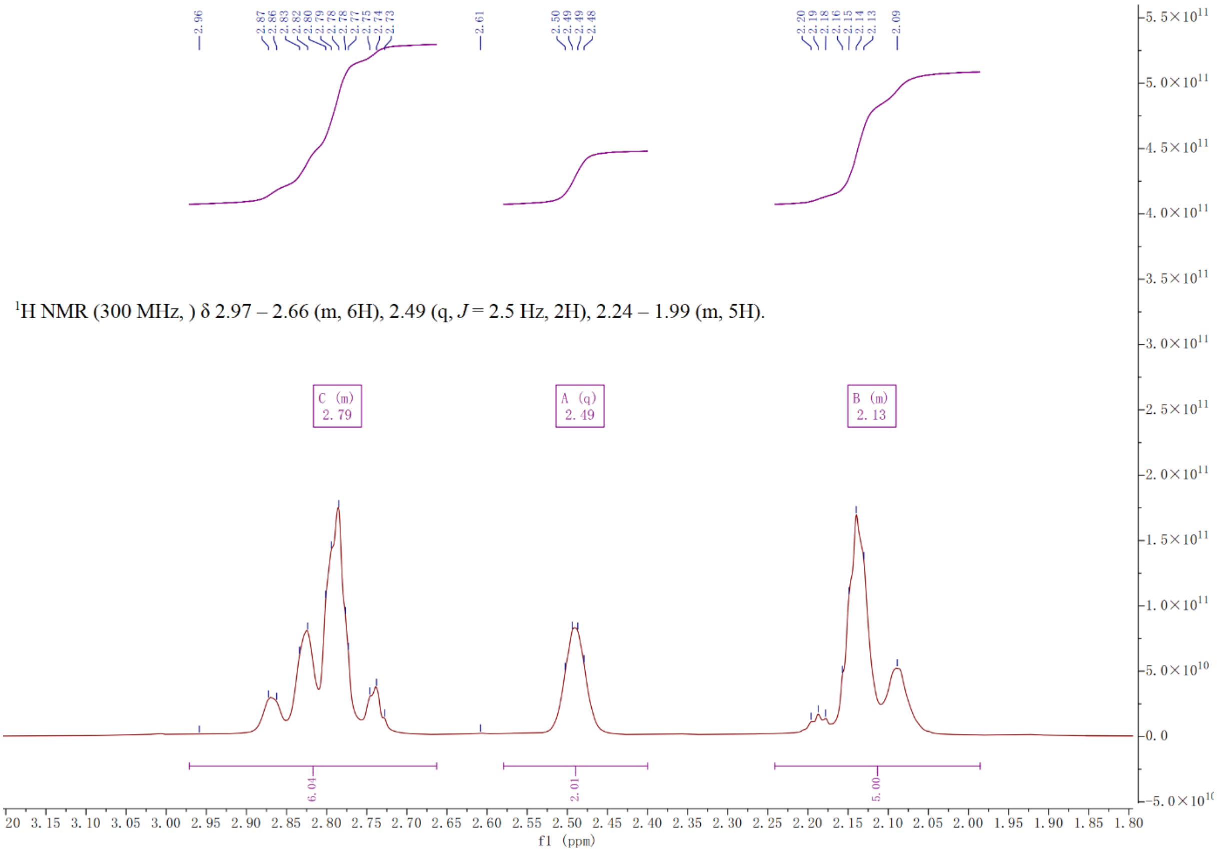

Figure S22. The ^{1}H NMR (300 MHz) spectrum of I-OA.

Table S1. The solvent selective effect on I-OA.

	Solvent	Space group	Physical characteristics
mono-I-OA	Petroleum ether, cyclohexane, toluene, ether, ethyl acetate, dichloromethane, acetone, or acetonitrile	$P2_1$	Ferroelectric
orth-I-OA	Ethanol, methanol, DMF, or DMSO	$Pna2_1$	Ferroelectric

Table S2. Crystal data and structure refinements for F-OA, Cl-OA, Br-OA, *mono*-I-OA and *orth*-I-OA.

Compound	F-OA	Cl-OA	Br-OA	*mono*-I-OA	*orth*-I-OA
Temperature	293 K	298 K	298 K	298 K	298 K
Formula	$C_{10}H_{13}FO$	$C_{10}H_{13}ClO$	$C_{10}H_{13}BrO$	$C_{10}H_{13}IO$	$C_{10}H_{13}IO$
Formula weight	168.10	184.65	229.1	276.10	276.10
Crystal system	Cubic	Monoclinic	Monoclinic	Monoclinic	Orthorhombic
Space group	$F\bar{4}3m$	$P2_1/c$	$P2_1/c$	$P2_1$	$Pna2_1$
a/Å	9.58500(10)	10.6686(2)	10.7341(3)	12.71715(12)	13.4179(6)
b/Å	9.58500(10)	7.39100(10)	7.5867(2)	12.34641(12)	7.1744(4)
c /Å	9.58500(10)	11.6893(2)	11.7586(3)	12.87331(11)	10.4139(4)
α/deg	90	90	90.0	90	90
β/deg	90	91.834(2)	92.061(3)	98.9306(9)	90
γ/deg	90	90	90	90	90
Volume /Å^3	880.60(3)	921.25(3)	956.96(4)	1996.75(3)	1002.50(8)
Z	4	4	4	8	4
F(000)	308.0	392.0	464.0	1072.0	536.0
Radiation λ (Cu Kα) (Å)	1.54178	1.54184	1.54178	1.54184	1.54184
2 Theta range (°)	26.298 to 149.646	8.292 to 151.268	8.242 to 151.778	6.95 to 151.694	13.196 to 150.752
Limiting indices	$-11 \le h \le 11$, $-11 \le k \le 11$, $-11 \le l \le 12$	$-13 \le h \le 12$, $-9 \le k \le 8$, $-14 \le l \le 14$	$-13 \le h \le 13$, $-6 \le k \le 9$, $-14 \le l \le 14$	$-11 \le h \le 15$, $-14 \le k \le 15$, $-16 \le l \le 16$	$-16 \le h \le 16$, $-8 \le k \le 8$, $-6 \le l \le 12$
Reflections collected /unique	119/3644	1826/5807	1898/5203	6888/15585	1347/3124
Data/restraints/parameter	119/0/15	1826/0/109	1898/0/110	6888/1/433	1347/1/109
GOF	1.181	1.071	1.066	1.121	1.086
Final R indices [$I > 2\sigma(I)$][a]	R_1 = 0.1518, wR_2 = 0.3048	R_1 = 0.0425, wR_2 = 0.1143	R_1 = 0.0394, wR_2 = 0.1010	R_1 = 0.0480, wR_2 = 0.1268	R_1 = 0.0446, wR_2 = 0.1203
R indices (all data)	R_1 = 0.1693, wR_2 = 0.3171	R_1 = 0.0475, wR_2 = 0.1194	R_1 = 0.0407, wR_2 = 0.1019	R_1 = 0.0523, wR_2 = 0.1308	R_1 = 0.0485, wR_2 = 0.1269
Largest diff. peak and hole, e/Å^{-3}	0.30/-0.19	0.29/-0.31	0.68/-0.60	0.93/-2.11	0.84/-0.66
Flack parameter	-0.5(6)	--	--	0.38(3)	0.129(18)

[a] $R_1 = \Sigma||F_o| - |F_c||/\Sigma|F_o|$, $wR_2 = [\Sigma w(F_o^2 - F_c^2)^2/\Sigma(F_o^2)^2]^{1/2}$
